# Attenuating Effect of Vitamin E against Silver Nano Particles Toxicity in Submandibular Salivary Glands

**DOI:** 10.3390/bioengineering8120219

**Published:** 2021-12-16

**Authors:** Mahmoud M. Bakr, Mahmoud M. Al-Ankily, Sara M. Shogaa, Mohamed Shamel

**Affiliations:** 1 School of Medicine and Dentistry, Griffith University, Gold Coast, QLD 4215, Australia; 2 Faculty of Dentistry, The British University in Egypt, Cairo 11837, Egypt; mahmoud.ankily@bue.edu.eg (M.M.A.-A.); sara.mostafa@bue.edu.eg (S.M.S.); mohamed.shamel@bue.edu.eg (M.S.)

**Keywords:** silver nanoparticles, nanotoxicology, submandibular gland, reactive oxygen species, vitamin E, antioxidants

## Abstract

Silver nanoparticles (AgNPs) are extensively used in many industries due to their superior antimicrobial properties. However, it is evident from many studies that AgNPs has cytotoxic potential through its effect on excessive formation of reactive oxygen species (ROS). The aim of this study was to examine the toxic effect of AgNPs on the submandibular salivary glands and the attenuating effect of vitamin E, as a natural antioxidant, against this toxicity. Thirty Albino rats were divided into 3 groups (*n* = 10): control group, AgNPs group receiving 2 mg/kg daily for 28 days, and AgNPs and vitamin E group receiving AgNPs the same as the previous group in addition to vitamin E at a dose of 100 mg/kg. Microscopic, ultrastructural, and cytokeratin immune-reactivity examination of the glands were performed. The AgNPs group showed noticeable degeneration in all structures of the gland as evident in the histological and ultrastructural examination. The AgNPs and vitamin E group revealed an improvement of the glandular elements. A significant increase in cytokeratin immune expression was found after comparison of both groups (*p* = 0.01). This current study shows that vitamin E has powerful antioxidant properties, which can combat the cytotoxic effect caused by AgNPs. Further studies are deemed necessary to confirm this finding using other immunohistochemical markers, such as myosin and E-cadherin.

## 1. Introduction

In the past few years, silver nanoparticles (AgNPs) have been extensively studied because of their wide commercial use in many products, such as household products [1,2], and in food industry [3,4,5]. Moreover, because of its powerful antimicrobial activity, these nanoparticles are recognized as a resourceful material for the drinking water purification industry [6,7]. Its high chemical stability and relatively low cost are among the main reasons for its popular use [8,9,10]. In addition to the above, AgNPs have multiple beneficial applications in medicine and dental biomaterials [11,12,13,14,15]. Advances in nanotechnology have allowed for the emergence of the term nanomedicine, which allowed for promising applications in laboratory research and biology including drug delivery and catheter coating [16,17,18,19,20,21,22,23,24]. However, many studies have reported potential toxicological effects of AgNPs. Those studies have proposed that AgNPs possess a possible cytotoxic effect through the generation of oxidative stress induced by the overproduction of reactive oxygen species (ROS) in various tissues and pathological conditions including cancer [25,26,27,28,29,30].

ROS are physiological byproducts of cellular biochemical reactions necessary for cellular metabolism. Under normal circumstances, different antioxidant enzyme systems are induced, and they serve to scavenge the formed ROS. However, any imbalance between the antioxidant systems and the ROS might lead to the formation of oxidative stress, which in turn causes damage to cellular organelles as well as to DNA and proteins, resulting in cell death [31,32]. Dietary antioxidants have been shown to be effective in decreasing the toxic effect of AgNPs [33]. Among the previously studied dietary antioxidants is vitamin E as a peroxyl radical scavenger, where it was demonstrated that it can decrease oxidative stress through attenuation of the oxidative cascade and its ability to neutralize ROS, thus protecting the affected organs [34,35,36,37,38,39].

Oral exposure of AgNPs is probably more common than any other routes of administration. AgNPs are used in the water industry for disinfecting drinking water of humans as well as animals. AgNPs can cross the gastrointestinal tract barrier and can be absorbed into the circulation, being translocated to different tissues, where AgNPs could exert their toxic effects [1]. Hence, in the current study, we studied the toxic effect of AgNPs on the submandibular salivary glands. The effect of a natural antioxidant, vitamin E, in combating AgNPs cytotoxicity was also evaluated.

## 2. Materials and Methods

### 2.1. Experimental Groups

Thirty adult male albino rats were used in this study (average weight 200 gms). Rats were housed in separate cages, four rats per dated cage, and kept under the supervision of a specialized veterinarian. Rats were kept under good ventilation and an adequate stable diet consisting of fresh vegetables, dried bread, and tap water ad libidum.

The animals were equally distributed into the following groups:

*Control group*: Rats received I.P injections with aqueous nitrate buffer at a dose of 2 mg/kg/B.W daily for 28 days [40].

*AgNPs group*: Rats received I.P injections with silver nano particles (AgNPs) at a dose of 2 mg/kg once daily for 28 days [40].

*AgNPs and Vitamin E group*: Rats received I.P injections with AgNPs at a dose of 2 mg/kg once daily the same as the previous group, in addition to administration of vitamin E by oral gavage at a dose of 100 mg/kg (149 IU) daily for 28 days [41].

### 2.2. Preparation and Characterization of the Silver Nanoparticles:

The rapid green method was used [42]. It was conducted in the nanotechnology lab in the Faculty of Science, South Valley University—Egypt. Pomegranate fruits were collected from the local market. Silver nitrate (AgNO_3_, >99.9%) was obtained. All glass-wares and pomegranate fruit were properly washed with de-ionized water and dried in an oven. Fruit peel extract (FPE) of pomegranate were used as a reducing agent for the development of silver nanoparticles. Properly washed 50 g of the fresh peels of the fruit were added to 250 mL of ultra-pure water in a 500 mL Erlenmeyer flask and boiled for 10–15 min. Whatman filter paper (No. 40) was used for filtration of the boiled material to prepare the aqueous fruit peel extract, which was used as such for metal nanoparticle synthesis.

Aqueous solution (1 mL) of silver nitrate solution was prepared and 50 mL of the metal (Ag) ion solution were reduced using 1.8 mL of FPE at room temperature for 5 min. Below this FPE quantity, the solution took more than 10 min to obtain a significant surface plasmon resonance (SPR) for the metal nanoparticles. Spectral analysis for the development of nanoparticles at different reaction conditions was conducted with UV-Vis spectroscopy using a Perkin-Elmer Lamda-45 spectrophotometer and X-ray powder diffraction (XRD). A Transmission Electron Microscope (TEM) JEM-1200 EX, JEOL 1010 was used for analysis of the size and shape of the developed nanoparticles. The average nanoparticle yield for AgNPs prepared from fruit peel extract (FPE) of pomegranate was 0.85 ± 0.4 mg of AgNPs per 1 g fruit peel tissue dry mass.

For TEM measurements, 3 µL of the sample solution were placed on copper grid, making a thin film of sample on the grid, and dried at room temperature for 15 min. Then, the extra sample was removed using the cone of a blotting paper and reserved in a grid box. The presence of elemental silver was determined using energy-dispersive X-ray analysis (EDX) with a Zeiss Evo 50 instrument. The pH of the solution was maintained by 1 N HCl and NaOH. The resulting solutions of the developed nanoparticles of silver were dried at 800 °C for X-ray powder diffraction measurement. X-ray powder diffraction data were acquired by a GBC EMMA diffractometer in the central laboratory at South Valley University.

The AgNPs produced in the current study were spherical, ranging between 10 and 55 nm, and cubical, ranging between 8 and 46 nm in size. The cubical AgNPs were predominant amongst the produced crystals. The spherical AgNPs (10 nm) were isolated and used in the current study.

All specimens were examined through:

(a) Hematoxylin & Eosin.
(b) Immunohistochemical examination.
(c) Electron microscope.

### 2.3. Image and Statistical Analysis

For descriptive (qualitative) purposes, the ant-cytokeratin staining reaction of the different submandibular salivary gland components of all experimental groups was scored as either:

(−) negative staining reactivity.

(+) weak positive staining reactivity.

(++) moderate positive staining reactivity.

(+++) strong positive staining reactivity.

Image analysis was performed via computer software Leica Quin 500 (LEICA Imaging Systems Ltd., Cambridge, UK) to evaluate the area percentage of cytokeratin immunostaining within the gland. The one-way ANOVA test was used to compare the mean area percent of cytokeratin immuno-expression between the specimens of experimental groups. Pair-wise comparison using the Post Hoc Tukey test was used to compare between each group. SPSS 25.0 for Windows (SPSS Inc., Chicago, IL, USA) was used for statistical analysis.

## 3. Results

### 3.1. Histological Examination

**Figure 1:**
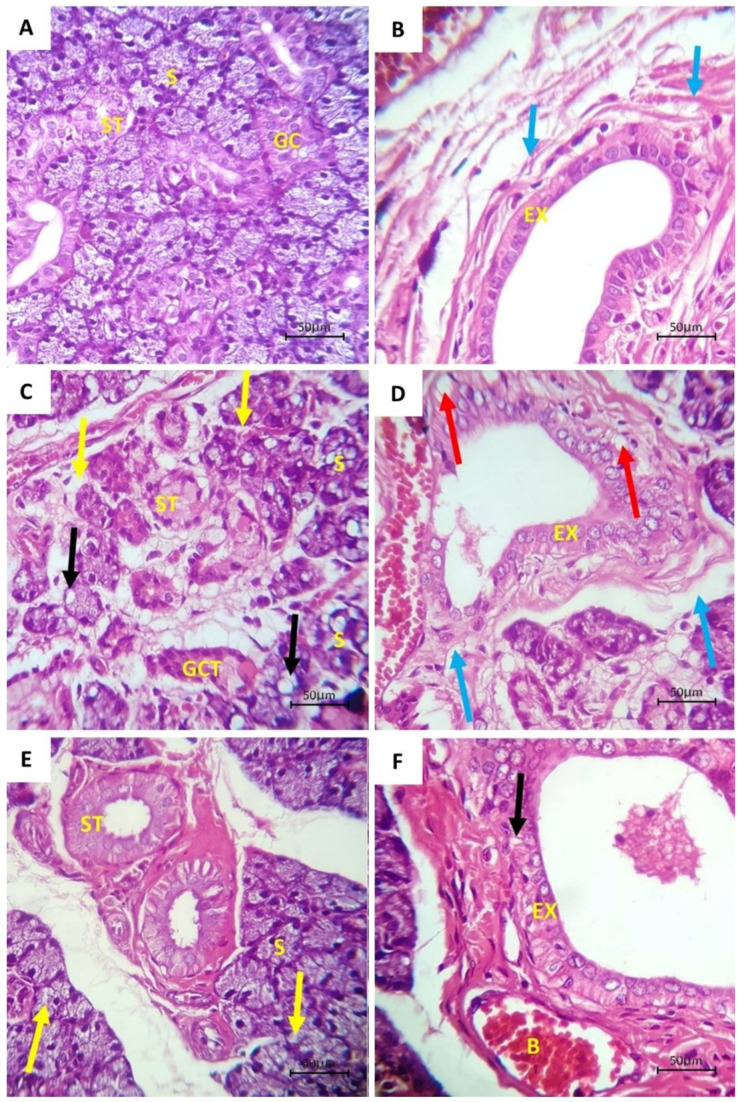
(**A**,**B**) Hematoxylin and eosin (H&E) staining in submandibular salivary glands of the control group displaying normal structures. Serous acini (S), granular convoluted tubule (GCT), striated duct (ST), and excretory duct (EX) surrounded by fibrous connective tissue stroma (blue arrows). (**C**,**D**) Hematoxylin and eosin (H&E) staining in the AgNPs group showing degenerated acini (red arrows), atrophic serous acini (S) with cytoplasmic vacuolation (black arrows), and wide interacinar spaces (yellow arrows). Vacuolated GCT and striated duct (ST) and excretory duct (EX) with thickened lining epithelium presenting extensive vacuolations (red arrows). Degeneration in the surrounding connective tissue (blue arrows). (**E**,**F**): Hematoxylin and eosin (H&E) staining in the vitamin E-treated group presenting typical serous acini (S), GCT with few cytoplasmic vacuoles (yellow arrows), and striated ducts with normal cell lining (ST). Dilated and congested blood vessels were observed (**B**) and the excretory duct (EX) shows relatively normal lining with slight vacuolations in some areas (black arrow) (original magnification ×400).

Control group: Histological examination of the glands of the control group showed a normal gland architecture, where serous acini and ductal systems all appeared with typical histological structures (Figure 1A,B). AgNPs group: Noticeable degeneration in the acini and duct was evident. There was apparent shrinkage and a reduction in the size and number of all the parenchymal elements including the acini and ducts. The acini were reduced and degenerated with a lot of cytoplasmic vacuolization. The GCTs showed a loss of regular configuration, while the striated ducts showed cytoplasmic vacuolization and loss of basal striations. The excretory ducts showed loss of the pseudo-stratification of their lining. Some other ducts showed thickening in their lining cells. Most ducts showed dilatation in their lumens with stagnant secretion. Connective tissue septa showed marked degeneration with dissociation of the collagen fibers and chronic inflammatory cell infiltrate (Figure 1C,D).

AgNPs and vitamin E group: The vitamin E-treated group showed histological sections, which showed an almost normal architecture with no or little cytoplasmic vacuolization. Striated ducts regained their basal striations, while the excretory ducts possessed cell lining with a pseudostratified columnar epithelium. Some of the ducts showed thickening in their epithelial lining. An increase in the fibrous CT content surrounding the ducts was sometimes recorded (Figure 1E,F).

### 3.2. Immunohistochemical Examination

**Figure 2:**
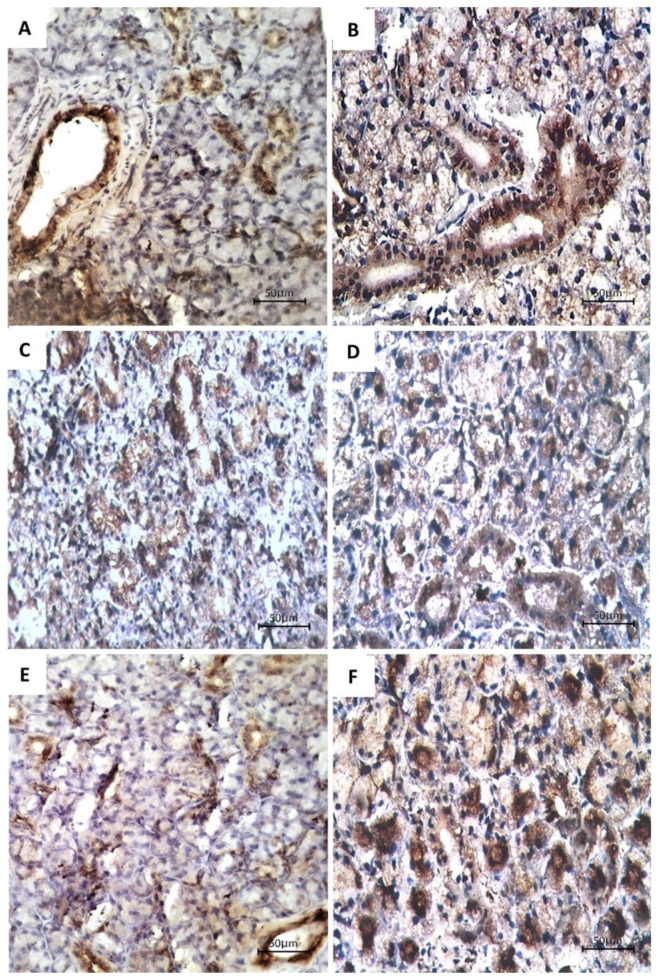
(**A**,**B**) Submandibular salivary gland from the control group showing a positive cytokeratin staining reaction of the entire gland components. (**C**,**D**) Submandibular salivary gland from the AgNPs group incubated with anti-cytokeratin monoclonal antibody showing a weak to moderate positive cytokeratin reaction in both the serous acinar cells and ductal cells. (**E**,**F**) Submandibular salivary gland from the rats after treatment with vitamin E and incubation with anti-cytokeratin monoclonal antibody displaying a moderate positive cytokeratin reaction of serous acinar cells, and a moderate to strong positive cytokeratin reaction in the ductal cells (original magnification ×400).

Cytokeratin immunoreactivity in the specimens of the control group showed a positive reaction (Figure 2A,B), while those of the AgNPs group revealed a weakly to moderately positive reaction (Figure 2C,D).

**Scheme 1:**
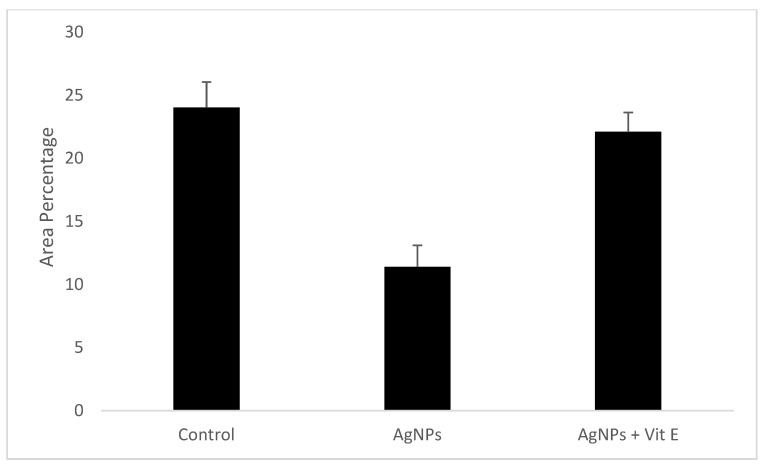
Mean area percent of cytokeratin immuno-expression of all groups.

The tissue sections of the AgNPs and vitamin E group unveiled a notable increase in cytokeratin immunoreactivity in the acini in comparison to the AgNPs group. On the other hand, a slight upsurge in immunoreactivity was shown in the ducts. However, the immunoreactivity appeared to be comparable to that of the control group (Figure 2E,F). The mean area percent of cytokeratin immunoreactivity in the submandibular salivary glands of all groups is summarized in Scheme 1.

Post Hoc Tukey test comparison between the groups revealed that the difference between the control group and AgNPs + vitamin E group was not significant (*p* = 0.055). However, a significant decrease was found between the AgNPs and control groups (*p* = 0.001), and a significant increase was also found between the AgNPs + vitamin E and AgNPs groups (*p* = 0.001) (Table 1 and Table 2).

### 3.3. Electron Microscope Examination

**Figure 3:**
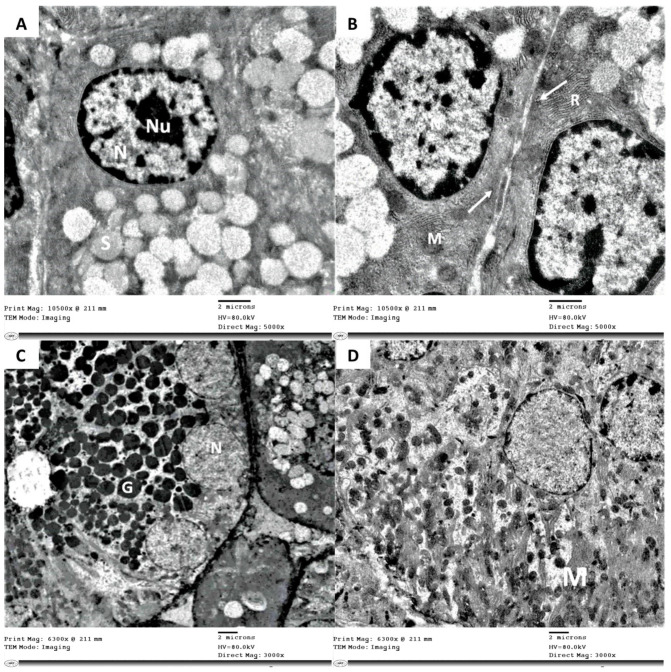
An electron micrograph of the submandibular gland from a control group showing: (**A**): Serous acinus with pyramidal-shaped cells and normal nucleus (Nu) and secretory granules (S), (**B**): Serous acini with normal cell to cell junction (arrows), normal mitochondria (M), and rough endoplasmic reticulum (R), (**C**): granular convoluted tubule with normal nucleus (N) and secretory electron-dense granules (G), (**D**): Striated duct with numerous radially arranged mitochondria (M).

**Figure 4:**
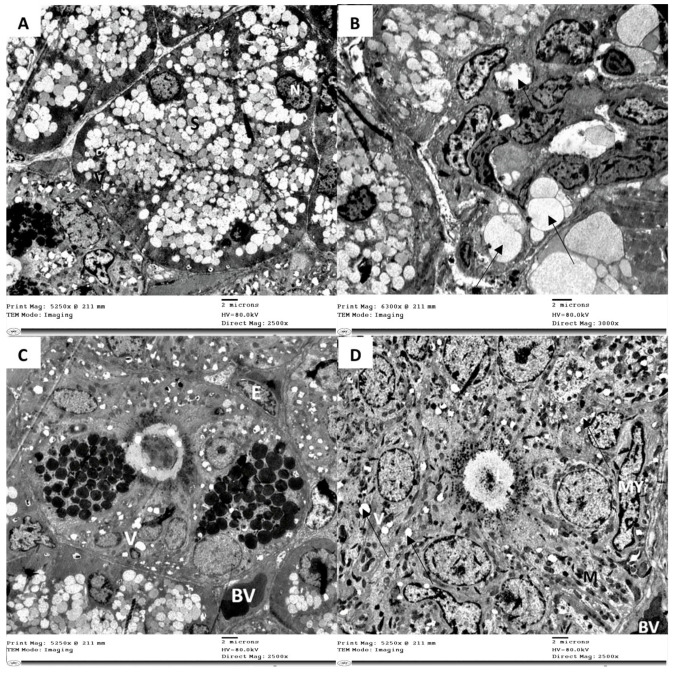
An electron micrograph of the submandibular gland from AgNPs showing: (**A**): acinar cells with irregular basally located nuclei (N), accumulation of electron-lucent secretory granules (S). Large and small cytoplasmic vacuolization was observed in the acinar cells (V), (**B**): Numerous vacuolization (arrows) in acinus and ducts, (**C**): granular convoluted tubule with a marked decrease in the size and number of their granular contents, pleomorphism of the granules, and great variation in their electron density. Cytoplasmic vacuolization of the duct cells (V), (**D**): Striated duct with degenerated mitochondria (M), vacuolization (arrows), and loss of basal folding.

**Figure 5:**
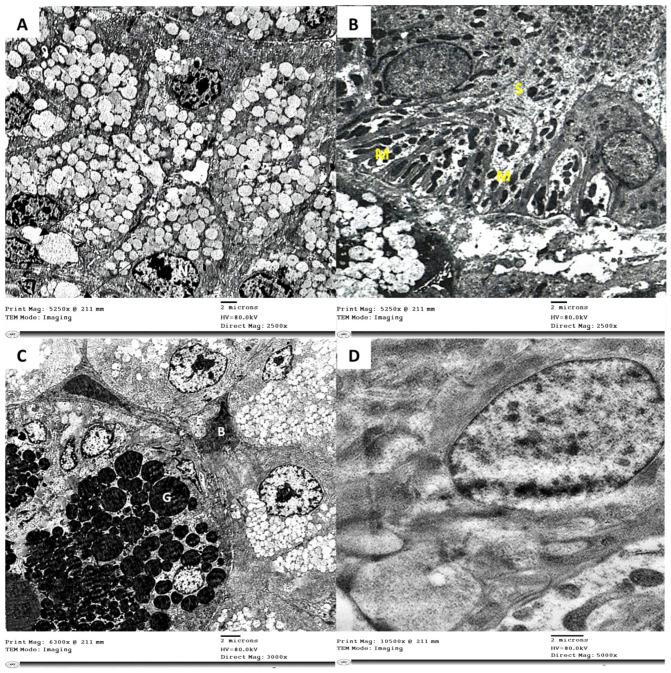
An electron micrograph of the submandibular gland from the vitamin E group showing (**A**): an almost normal acinus structure with basally located nucleus (N), and myoepithelial cell (M), few vacuolization (arrows) can be observed, (**B**): striated duct with almost normal basal striations, moderate number of elongated radially arranged mitochondria (M), and electron dense secretory vesicles (S); (**C**): granular convoluted tubules lined with columnar cells with a lot of electron-dense granules (G). (**D**): Striated duct with numerous radially arranged mitochondria (M).

The electron microscopic examination of the submandibular salivary glands of the control group showed normal ultrastructural elements (Figure 3). While the examination of glands of the AgNPs group showed severe ultrastructural changes of the secretory portions and ducts as well as the connective tissue stroma. Acini and ducts appeared to be degenerated, where the most frequently observed feature was the appearance of many cytoplasmic vacuolations. Organelles, such as nuclei, mitochondria, and rough endoplasmic reticulum (RER), showed massive degeneration (Figure 4). Glands of the group treated with vitamin E showed noticeable enhancement of the acini and ducts with insignificant changes (Figure 5).

## 4. Discussion

Several studies have reported the cellular toxicity caused by the increased formation of ROS derived from AgNPs exposure despite their widespread usage in commercial consumer products including drug delivery systems, medical devices, and supplies [43,44]. In fact, AgNPs toxicity has been reported in many tissues, such as liver, kidneys, intestine, and reproductive systems [45,46,47,48]. Oral tissue toxicity following AgNPs exposure has been previously reported in salivary glands and lingual mucosa [29,49]. In the current study, evidence of AgNPs toxicity in the submandibular salivary glands was evident through histological, immunohistochemical, and ultrastructural results.

In the present study, the histological results of the AgNPs group showed obvious deterioration of the gland, evident by the variable degrees of degenerative changes involving most of the acinar and ductal cells. The most noticeable observation was the frequent formation of cytoplasmic vacuolization in the serous acini as well as in the intraglandular ducts. These histological changes were also observed by several studies where rats were intoxicated with AgNPs [29,50]. These findings were explained by Asharni et al. (2009) [51], who found that AgNPs can cause DNA and chromosomal abnormalities. This damage was triggered indirectly by AgNPs, which increases the ROS production, which induces mitochondrial damage and sequentially weakens energy-dependent DNA repair mechanisms. We also suggest that the histological changes that were observed in the GCTs and the striated ducts may have occurred due to the decrease in the metabolic activity of the cells or the reduction in protein synthesis due to the accumulation of AgNPs in the ductal cells as well as their production of free radicals [52].

In the current study, it was observed that the excretory ducts showed a loss of pseudo stratification. These results are similar to those obtained by Shamel et al. (2021) [29], where they found that the excretory ducts of the submandibular salivary glands of albino rats appeared vacuolated and degenerated and lost their pseudo stratification after administration of AgNPs. The ultrastructural results of the AgNPs group support the previously mentioned histological results, which showed severe ultra-structural changes of the secretory portions and ducts as well as the CT stroma. These results agreed with several studies where the ultrastructural results of the parotid glands of albino rats after injection of AgNPs displayed strong cytoplasmic vacuolization and numerous intranuclear and mitochondrial vacuoles of various sizes [50].

Our findings could be clarified by Ma et al. (2015) [53], who reported that AgNPs induce mitochondrial membrane depolarization and subsequently mitochondrial damage. Similarly, a recent study reported that AgNPs can induce cell apoptosis by the mitochondrial-dependent pathway [54]. This study revealed that AgNPs exposure leads to the generation of ROS associated with oxidative and cytotoxic events and subsequently induction of apoptosis [54]. Overproduction of ROS and the activation of oxidizing enzymes lead to cytoskeletal, cell membrane and mitochondrial damage, and germ cell-specific apoptosis [55,56]. All the previous hypotheses can explain the previously stated histological and ultrastructural changes in the foundations of the submandibular salivary glands.

Immunohistochemical localization of cytokeratin in the glands of rats of the AgNPs group with their distorted glandular elements represented a generalized decrease in their staining reactivity to cytokeratin in comparison to the control group, which was statistically significant. This difference regarding the immunohistochemical staining distribution of cytokeratin proteins was the result of atrophic and degenerative changes in the acinar and ductal cells, which were confirmed histologically and ultra-structurally. The reduction of cytokeratin immuno-staining in the glands of the AgNPs group rats compared to the control group signifies a decrease in the cytoplasmic content of cytokeratins, which have a vital role in many cellular interactions, such as cell to cell contact. Cytokeratin also has a role in anchoring the nucleus within the cell and in forming the structure of vital cell organelles, such as the Golgi apparatus [57]. Thus, any disturbance in cytokeratins in the salivary glands might lead to distortion of acini, defective cellular junctions leading to wide intercellular junctions, distorted organelles, and subsequently cell degeneration [58,59].

On the other hand, vitamin E at a dose of 100 mg/kg for 28 days significantly improved the salivary gland structure both histologically and ultra-structurally. The vitamin E-treated group showed signs of recovery where an almost normal texture with no or little cytoplasmic vacuolization was observed in the glandular elements. These results could be explained by the fact that vitamin E is a powerful antioxidant that constrains the production of ROS molecules when fat undergoes oxidation and during the propagation of free radical reactions. It has been found that vitamin E mainly inhibits the production of new free radicals, and at the same time neutralizes the existing free radicals. The previous data could explain the attenuating role of vitamin E against AgNPs toxicity, which is mainly caused by elevated ROS formation [60].

Another explanation for the role of vitamin E is that it provides greater stability to the cell by increasing the lipid content of cell membranes, thus allowing for a more stabilized membrane [61]. Therefore, vitamin E helps membrane repair by averting the formation of oxidized phospholipids that might interfere with the membrane fusion events [62]. On the other hand, there was no significant difference in cytokeratin expression between the vitamin E group and the control group. The cytokeratin expression showed moderate staining immunoreactivity, indicating the improvement noted in the structural and ultrastructural results.

The AgNPs dose used in the current study was according to ElMahdy et al., 2015 [40], who reported that doses ranging from 1–4 mg/kg for 28 days were capable of inducing histopathological alterations through oxidative stress and the creation of chromosomal aberrations in bone marrow cells due to the genotoxicity of AgNPs. Furthermore, the dose used in the current study was relevant to potential human exposure. In addition to the above, Lytvynenko et al., 2017 [63] found similar results in the female reproductive system with AgNPs doses of 2 and 4 mg/kg. A more recent study used the same dose of AgNPs (2 mg/kg) to induce toxicity and degeneration in the parotid salivary gland, which was confirmed by nuclear changes, cytoplasmic vacuolations, and partial blockage in the ductal lumen [50].

The production of size- and shape-specific AgNPs is highly important in usage for different applications due to their unique optical and catalytic properties [64]. It was proven that smaller sized AgNPs showed higher antibacterial properties as well as higher toxicity [65]. Furthermore, the plate-shaped AgNPs had higher antibacterial properties than the spherical and cubic AgNPs [65]. The high variability in AgNPs is not only size and shape dependent; other factors including PH and temperature affect the properties of the produced AgNPs [66]. On the other hand, another study demonstrated that AgNPs’ size did not affect the toxicologic parameters and tissue distribution [67]. All of the above could explain the variability in the results obtained from different studies due to the inconsistency in the physical properties of AgNPs used in each individual study.

The process of functionalizing the AgNPs is important for their long-term stability [68] and biophysical properties [69]. The functional layer of nanoparticles plays an important role in antibacterial properties [70,71]. Furthermore, functionalized AgNPs allowed for more DNA damage when compared to non-functionalized AgNPs, which had less surface area availability and access to cell organelles [72]. In addition to the above, functionalizing AgNPs results in modulation of their bioactivity features including oxidative stress and cellular uptake [73]. Non-functionalized AgNPs allow interaction with different molecules including glucose, which could further alter its properties [74]. Despite functionalized AgNPs having unique properties that are useful in scientific and medical applications including food packaging [75], improvement of antibiotics [76,77], and anti-platelet activity [78], the AgNPs used in the current study were not functionalized to exclude any potential effects of the surface coating and model the human consumption scenario.

The current study serves as a pilot study for further investigations related to AgNPs toxicity on different tissues including tongue papillae, gingiva, alveolar bone, and the periodontal ligament. There are some limitations within the scope of the current study and further research is needed to quantify the amount of ROS produced as a result of AgNPs toxicity and quantifying the amount of damage in the submandibular salivary glands through measurement of the apoptotic rate using flow cytometry and/or apoptotic cell markers. Furthermore, the results obtained from the current study could be compared to the effects of other antioxidants, such as vitamin A and vitamin C, in counteracting the AgNPs toxicity.

## 5. Conclusions

Within the limitations of the current study, we conclude that AgNPs are capable of causing degeneration in the parenchymal elements of the submandibular salivary glands. This degeneration was evident by the histological and ultrastructural results as well as immunohistochemical localization of cytokeratin. Interestingly, vitamin E ameliorated AgNPs toxicity and restored the degeneration caused by silver nanoparticles (AgNPs) in submandibular salivary glands. Further studies are deemed necessary to confirm this finding using other immunohistochemical markers, such as myosin and E-cadherin, apoptotic markers including Bcl-2, P27, and P53, as well as quantifying apoptosis by using flow cytometry.

## Figures and Tables

**Table 1:** Area percentage of cytokeratin immuno-reactivity in different groups.

	Control	AgNPs	AgNPs + Vit E	*p*-Value
**Minimum**	19.48	7.95	18.42	0.001
**Median**	24.76	11.48	22.55	
**Maximum**	25.87	14.11	23.67	
**Mean**	24.03	11.41	22.1	
**Standard Deviation**	2.02	1.70	1.54	
**Standard Error**	0.74	0.6	0.916	

**Table 2:** Tukey’s Post Hoc Test for cytokeratin immuno-reactivity in different groups.

Tukey’s Multiple Comparison	Significance (*p* > 0.05)	Summary
**Control vs. AgNPs**	0.001	Significant
**Control vs. AgNPs + Vit E**	0.055	Not significant
**AgNPs vs. AgNPs + Vit E**	0.001	Significant

## Data Availability

The data presented in this study are available on request from the corresponding author.
